# Enduring differential patterns of neuronal loss and myelination along 6-month pulsatile gonadotropin-releasing hormone therapy in individuals with Down syndrome

**DOI:** 10.1093/braincomms/fcaf117

**Published:** 2025-03-22

**Authors:** Michela Adamo, Mihaly Gayer, An Jacobs, Quentin Raynaud, Raphael Sebbah, Giulia di Domenicantonio, Adeliya Latypova, Nathalie Vionnet, Ferath Kherif, Antoine Lutti, Nelly Pitteloud, Bogdan Draganski

**Affiliations:** Department of Endocrinology, Diabetes and Metabolism, Lausanne University Hospital, University of Lausanne, CH-1011 Lausanne, Switzerland; Laboratory for Research in Neuroimaging LREN, Centre for Research in Neurosciences, Department of Clinical Neurosciences, Lausanne University Hospital, University of Lausanne, CH-1011 Lausanne, Switzerland; Department of Endocrinology, Diabetes and Metabolism, Lausanne University Hospital, University of Lausanne, CH-1011 Lausanne, Switzerland; Laboratory for Research in Neuroimaging LREN, Centre for Research in Neurosciences, Department of Clinical Neurosciences, Lausanne University Hospital, University of Lausanne, CH-1011 Lausanne, Switzerland; Department of Endocrinology, Diabetes and Metabolism, Lausanne University Hospital, University of Lausanne, CH-1011 Lausanne, Switzerland; Laboratory for Research in Neuroimaging LREN, Centre for Research in Neurosciences, Department of Clinical Neurosciences, Lausanne University Hospital, University of Lausanne, CH-1011 Lausanne, Switzerland; Laboratory for Research in Neuroimaging LREN, Centre for Research in Neurosciences, Department of Clinical Neurosciences, Lausanne University Hospital, University of Lausanne, CH-1011 Lausanne, Switzerland; Department of Endocrinology, Diabetes and Metabolism, Lausanne University Hospital, University of Lausanne, CH-1011 Lausanne, Switzerland; Laboratory for Research in Neuroimaging LREN, Centre for Research in Neurosciences, Department of Clinical Neurosciences, Lausanne University Hospital, University of Lausanne, CH-1011 Lausanne, Switzerland; Laboratory for Research in Neuroimaging LREN, Centre for Research in Neurosciences, Department of Clinical Neurosciences, Lausanne University Hospital, University of Lausanne, CH-1011 Lausanne, Switzerland; Department of Endocrinology, Diabetes and Metabolism, Lausanne University Hospital, University of Lausanne, CH-1011 Lausanne, Switzerland; Laboratory for Research in Neuroimaging LREN, Centre for Research in Neurosciences, Department of Clinical Neurosciences, Lausanne University Hospital, University of Lausanne, CH-1011 Lausanne, Switzerland; Neurology Department, Max Planck Institute for Human Cognitive and Brain Sciences, D-04103 Leipzig, Germany; Department of Neurology, Inselspital, University of Bern, CH-3010 Bern, Switzerland; University Institute for Diagnostic and Interventional Neuroradiology, Inselspital, University of Bern, CH-3010 Bern, Switzerland

**Keywords:** trisomy 21, magnetic resonance imaging, multi-parameter mapping, morphometry, brain tissue properties

## Abstract

Despite major progress in understanding the impact of the triplicated chromosome 21 on the brain and behaviour in Down syndrome, our knowledge of the underlying neurobiology in humans is still limited. We sought to address some of the pertinent questions about the drivers of brain structure differences and their associations with cognitive function in Down syndrome. To this aim, in a pilot magnetic resonance imaging (MRI) study, we monitored brain anatomy in individuals with Down syndrome receiving pulsatile gonadotropin-releasing hormone (GnRH) therapy over 6 months in comparison with typically developed age- and sex-matched healthy controls. We analysed cross-sectional (Down syndrome/healthy controls *n*  *=* 11/27; Down syndrome—2 females/9 males, age 26.7 ± 5.0 years old; healthy controls—8 females/19 males, age 24.1 ± 2.5 years old) and longitudinal (Down syndrome/healthy controls *n*  *=* 8*/*13; Down syndrome—1 female/7 males, age 26.4 ± 5.3 years old; healthy controls—4 females/9 males, 24.7 ± 2.2 years old) relaxometry and diffusion-weighted MRI data alongside standard cognitive assessment. The statistical tests looked for cross-sectional baseline differences and for differential changes over time between Down syndrome and healthy controls. The *post hoc* analysis confined to the Down syndrome group, tested for potential time-dependent interactions between individuals’ overall cognitive performance and associated brain anatomy changes. The brain MRI statistical analyses covered both grey and white matter regions across the whole brain allowing for investigation of regional volume, macromolecular/myelin and iron content, additionally to diffusion tensor and neurite orientation and dispersion density characterization across major white matter tracts. The cross-sectional analysis showed reduced frontal, temporal and cerebellar volumes in Down syndrome with only the cerebellar differences remaining significant after adjustment for the presence of microcephaly (*P*_family-wise-corrected_ < 0.05). The volume reductions were paralleled by decreased cortical and subcortical macromolecular/myelin content confined to the cortical motor system, thalamus and basal ganglia (*P*_family-wise-corrected_ < 0.05). All major white matter tracts showed a ubiquitous mean diffusivity and intracellular volume fraction reduction contrasted with no differences in magnetization transfer saturation metrics (*P*_family-wise-corrected_ < 0.05). Compared with healthy controls over the same period, Down syndrome individuals under GnRH therapy showed cognitive improvement (Montreal Cognitive Assessment from 11.4 ± 5.5 to 15.1 ± 5.6; *P* < 0.01) on the background of stability of the observed differential neuroanatomical patterns. Despite the lack of adequate Down syndrome control group, we interpret the obtained cross-sectional and longitudinal findings in young adults as evidence for predominant neurodevelopmental neuronal loss due to dysfunctional neurogenesis without signs for short-term myelin loss.

## Teaser

Adamo *et al*. investigate brain tissue properties and their associations with cognitive function in individuals with Down syndrome receiving pulsatile gonadotropin-releasing hormone therapy. Over 6 months, quantitative magnetic resonance imaging reveals stable differential patterns of neuronal loss and myelination in comparison with a healthy control group.

## Graphical Abstract

**Graphical Abstract:**
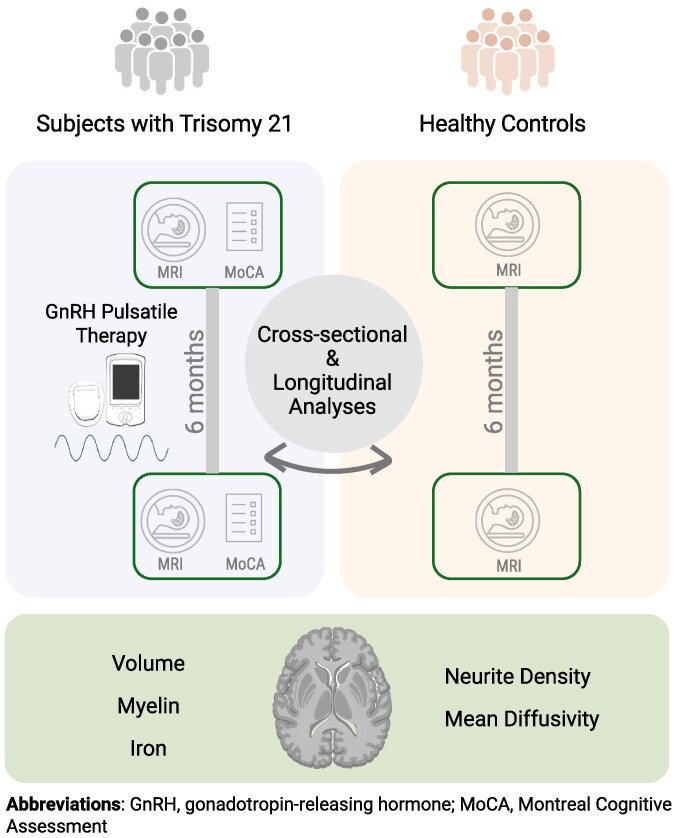

## Introduction

Down syndrome (DS), one of the most frequent neurodevelopmental disorders,^1^ caused by an extra copy of the chromosome 21, presents with variable degrees of behavioural, cognitive and motor impairments.^2^ The interaction between neurodevelopmental and neurodegenerative pathology, including deficient neurogenesis and synaptogenesis,^3-6^ impaired myelination,^7,8^ amyloid precursor protein overproduction^9^ and immuno-metabolic dysfunction,^10^ to name but a few, provides a framework for interpreting the observed interindividual and age-dependent variability in the clinical presentation of DS. Given the amyloid-related pathology and the observed cognitive decline in the context of increased life expectancy in DS,^9,11,12^ researchers also consider DS as a genetic model of Alzheimer’s disease.^13^

Despite major advances in understanding the neurobiology of DS-induced processes based on animal models,^14-16^ we still lack a detailed knowledge about the trajectories of corresponding brain changes in humans. To date, the number of published post-mortem and computational anatomy studies analysing statistically *in vivo* magnetic resonance imaging (MRI) data remains low (for review, see Neale *et al*.^17^). The reported differences in brain structure comprise a wide range of cortical and subcortical regions, with consistent findings of cerebellar and hippocampal volume reduction, contrasted by conflicting morphometry findings in frontal, parietal and temporal cortical areas.^18-30^ Such discrepancies may arise from the wide age range of the analysed cohorts, spanning over several decades from early childhood to young adulthood or from adolescence to old age (see Supplementary Table 1 and Neale *et al*.^17^). This raises questions about potential interaction effects between DS-related pre- and postnatal neurodevelopmental aberrations in children and the progressing Alzheimer’s disease neuropathology in adults. Another factor contributing to the inconsistent results is the variable use of adjustments for one of the cardinal anthropometric features of DS—brachy-/microcephaly, when testing for localized brain anatomy differences. The proxy for head size—total intracranial volume (TIV), represented by the sum of grey matter (GM), white matter (WM) and cerebrospinal fluid volumes, is either not used at all or used only to adjust (subcortical) volume measurements.^29^ However, the surface-based morphometrics of interest, cortical thickness and surface area, present a different and topologically variable proportion of shared variance with TIV compared with volume-based estimates.^31,32^ The question of how to appropriately adjust for global brain metrics in whole-brain surface-based analyses is particularly relevant in DS studies, given the reported findings of cortical thickness increases largely overlapping with reduced GM volume and surface area.^33^

Furthermore, a previous study provided an additional insight into the reason behind the conflicting computational brain anatomy results, by showing that at least 40% of cortical thickness differences in DS overlap with GM–WM contrast decreases.^30^ As demonstrated empirically, such contrast differences, driven by variations in the underlying brain tissue components, myelin, iron and water content,^34,35^ can lead to spurious morphometric findings in studies relying on T1-weighted MRI.^36^ Quantitative MRI techniques, based on biophysical models and subject-specific correction for bias field inhomogeneities, help minimizing the likelihood of spurious morphometry findings and facilitate the neurobiological interpretation of the obtained statistical results.^35^ Due to their inherently low levels of spatial and temporal bias, quantitative MRI data are also particularly well suited for longitudinal studies.^37^ Our established multi-parameter mapping protocol incorporates quantitative B0- and B1-field inhomogeneity correction, maps of longitudinal (R1) and transverse (R2*) relaxation rates, magnetization transfer saturation (MTsat) and effective proton density (PD*) indicative for iron, myelin or unbound tissue water content in the brain tissue.^35,36^

Another obstacle to a detailed understanding of the brain anatomy trajectories in DS was the traditional segregation of computational anatomy studies focusing on either the brain’s GM or WM, with the first being the domain of T1-weighted MRI, whereas the latter typically relies on diffusion-weighted imaging (DWI). While early volumetric studies confirmed the notion of DS-related WM volume decreases, predominantly in frontal and temporal areas,^20,38^ the handful DWI investigations employing tensor model estimates of fractional anisotropy (FA) and mean diffusivity (MD) revealed signal differences in the corpus callosum and all main long association fibres (for review, see Saini *et al*.^39^). Similar to the GM morphometry reports, the reviewed nine diffusion tensor imaging (DTI) studies cover a broad age range spanning from toddlers, children and young adults^40-42^ to adulthood^43-46^ and focus on differentiating the effects of Alzheimer’s disease comorbidity in the elderly.^46-48^ Three additional DTI studies published after the mentioned review investigated correlations between diffusion tensor indices and sleep characteristics,^49^ amyloid burden^50^ or plasma neurofilament light^51^ in individuals in their fourth or fifth decades of life, when Alzheimer’s disease pathology is pervasive.^13,52^ Given the empirical evidence for genome-wide perturbations in genes associated with oligodendrocyte differentiation and myelination from mid-foetal development to adulthood with diverging temporal trajectories in frontal and cerebellar cortex,^8^ the need for detailed characterization of myelin and neurite contributions to *in vivo* MRI measurements becomes clear. Here, in contrast to the DTI, which provides summary statistics of the diffusion signal without incorporating tissue-specific properties, we used neurite orientation density and dispersion imaging,^53^ a biophysical model to parametrize the diffusion signal as a function of intracellular volume fraction (ICVF),^54,55^ interpreted as indicative for neurite density. When combined with the myelin content indices derived from the independent R1 and MTsat multi-parameter mappings data set, this strategy offered the interpretational specificity required to characterize tissue microstructure, which is not achievable with DTI parameters alone (for review, see Kamiya *et al*.^55^).

Given our promising results on the effects of gonadotropin-releasing hormone (GnRH) pulsatile therapy on cognition in DS,^56^ we used relaxometry-based multi-parameter mappings alongside DWI-derived tensor and neurite orientation density and dispersion imaging indices to gain detailed insights into the tissue microstructural underpinnings of morphometry differences between DS and healthy controls across brain’s GM and WM. To minimize the potential confounding effects of progressing Alzheimer’s disease pathology, we focused on a narrow age range of participants (20–30 years) to study cross-sectional and longitudinal brain changes following 6 months of GnRH pulsatile therapy. Building on the trisomy-mouse model findings, we hypothesized that GnRH therapy would be associated with myelin changes in limbic cortical and subcortical areas, as well as in the corresponding WM projections.

Addressing the less frequently discussed topic of MRI data quality in DS studies, where up to 10–20% of acquired images have been discarded in previous work based on visual inspection criteria, we applied a dedicated prospective motion correction technique. This approach enabled the acquisition of artefact-free data and provided a quantitative metric of image quality.^57^

## Materials and methods

### Participants

For the cross-sectional analysis of cortical and subcortical GM, we included 38 participants: 11 individuals with DS (2 females/9 males; age 26.7 ± 5.0 years old) and 27 typically developed controls (8 females/19 males; age 24.1 ± 2.5 years old). The longitudinal GM analysis comprised 21 participants—8 individuals with DS (1 female/7 males; age 26.4 ± 5.3 years old) and 13 typically developed controls (4 females/9 males; 24.7 ± 2.2 years old). Both the cross-sectional and longitudinal analyses of the WM included 19 age- and sex-matched participants: 7 individuals with DS (1 female/6 males; age 26.9 ± 5.8 years old) and 12 typically developed controls (4 females/8 males; age 24.4 ± 2.4 years old). The longitudinal study involved individuals with DS who received GnRH therapy for 6 months as previously detailed^56^ and control subjects who underwent two MRIs 6 months apart without receiving any intervention. The typically developed controls were drawn from a historical cohort of age- and sex-matched participants in pilot projects with longitudinal data covering a 6-month period. The discrepancy in participant numbers reported for the GM and WM analyses resulted from missing or corrupted data. Genetic testing confirmed the presence of an extra copy of chromosome 21 in all DS individuals. For the current prospective open-label pilot study, we acquired only data from the treatment group, thus lacking a control group and blinding for therapy (ClinicalTrials.gov, NCT04390646). The general cognitive abilities were assessed with the Montreal Cognitive Assessment (MoCA) using two different versions at baseline and follow-up after 6 months to avoid practice effects. We summarized all demographic and cognitive data in Table 1. All participants or their legal representatives signed an informed consent approved by the local Ethics Committee.

**Table 1:** Demographic characteristics of study participants

		DS	CTR	Test	*P*-value
Grey matter					
Cross-sectional study	*N*	11	27		
	Sex	2F/9M	8F/19M	*χ* ^2^	0.748
	Age (years ± SD)	26.7 ± 5.0	24.1 ± 2.5	Unpaired *t*-test	0.130
Longitudinal study	*N*	8	13		
	Sex	1F/7M	4F/9M	*χ* ^2^	0.669
	Age (years ± SD)	26.4 ± 5.6	24.4 ± 2.4	Unpaired *t*-test	0.357
White matter					
Cross-sectional and longitudinal studies	*N*	7	12		
	Sex	1F/6M	4F/8M	*χ* ^2^	0.712
	Age (years ± SD)	26.9 ± 5.8	24.4 ± 2.4	Unpaired *t*-test	0.321
	MoCA at baseline	11.4 ± 5.5			
	MoCA 6M GnRH therapy	15.1 ± 5.6		Paired *t*-test (BL versus 6 M)	0.006

### MRI data acquisition

The MRI data were acquired on a 3-T whole-body MRI system (Prisma, Siemens Healthcare, Erlangen, Germany) using a 64-channel radiofrequency (RF) head-receive coil and RF body-transmit coil. The whole-brain relaxometry protocol comprised 3D multi-echo FLASH data sets with predominantly proton density-weighting [repetition time (TR) = 24.5 ms, flip angle *α* = 6°], T1-weighting (TR/*α* = 24.5 ms/21°) and magnetization transfer-weighting (TR/*α* = 24.5 ms/6°) contrast according to the previously published protocol.^58^ We acquired multiple gradient echoes with alternating readout polarity at six equidistant echo times (TE) between 2.34 and 14.04 ms for the magnetization transfer-weighting acquisitions and at eight equidistant TE between 2.34 and 18.72 ms for the proton density-weighting and T1-weighting acquisition yielding data with 1.5 × 1.5 × 1.5 mm voxel size, GRAPPA factor 2 in phase-encoding direction, 6/8 partial Fourier in partition direction and non-selective RF excitation.

The DWI protocol comprised a 2D echo-planar imaging with TR = 7400 ms, TE = 69 ms, parallel GRAPPA acceleration factor = 2, field of view = 192 × 212 mm, voxel size = 2.2 × 2.2 × 2.2 mm, matrix size = 96 × 106, 70 axial slices and 118 gradient directions (15 at *b* = 650 s/mm^2^, 30 at *b* = 1000 s/mm^2^, 60 at *b* = 2000 s/mm^2^ and 13 at *b* = 0 interleaved throughout the acquisition).

To map the spatial distribution of the RF transmit field B1+, we acquired data using the 3D echo-planar imaging spin-echo/stimulated echo method (field of view 256 × 192 × 192 mm, matrix 64 × 48 × 48, TR = 500 ms).^59,60^ B0-field mapping data were also acquired using a 2D dual-echo FLASH sequence (slice thickness = 2.5 mm, TR = 7000 ms, TE1/TE2 = 4.92/7.38 ms, *α* = 80°, BW = 290 Hz/pixel) to correct for the geometric distortions of the echo-planar imaging data.

To reduce potential image degradation due to patient movement during MRI data acquisition, we used a prospective correction system (KinetiCor, HI, Honolulu). The position of the patients’ head during the MRI acquisitions, monitored by an optical camera attached to the scanner bore, was transferred to the MRI scanner for real-time adjustment.^61,62^ To further mitigate the impact of head motion on data quality, data acquisition was automatically suspended during periods of excessive head movement (threshold for the motion degradation index Mew_th_ set at ΔMew_th_ = 3.5e^−4^ mm/s) as described previously.^57^

### Automated parcellation and sampling in multi-parameter maps

From the acquired raw MRI data, we calculated whole-brain maps of MTsat, effective longitudinal relaxation rate (R1), proton density (PD*) and transverse relaxation rate (R2*), using the VBQ implementation of the hMRI toolbox^63,64^ in SPM12 (Wellcome Centre for Human Neuroimaging, UCL, London, UK) running under MATLAB_R2021 (MathWorks, Natick, MA, USA). We corrected the derived maps for the inhomogeneities effects of the RF transmit field using the dedicated B1+-mapping data.^65,66^ We estimated individuals’ GM volume maps using SPM12s multi-channel ‘unified segmentation’ of MTsat and PD* maps^67^ with enhanced tissue priors that provide superior detection of the thalamus and basal ganglia.^68^ The sum of GM, WM and cerebrospinal fluid provided a TIV estimate, which is a well-established proxy of head size in individuals with copy number variations.^69^ Finally, we calculated 127 regional averages of volume, MTsat, R1 and R2* values across cortical and subcortical GM areas using the factorization-based image labelling.^70^

### DWI pre-processing and tract-based sampling

We pre-processed the DWI data with Mrtrix3^71^ including denoizing^72^ and Gibbs ringing artefacts removal.^73^ For Eddy current correction and data realignment due to subjects’ movement, we used the FSL 5.0 EDDY tool,^74^ while for the echo-planar imaging susceptibility distortions, we applied the acquired B0 maps in the SPM12s FieldMap toolbox.^75^ The diffusion maps were spatially aligned to the MTsat images using SPM12s rigid body registration.

We estimated DTI MD and FA maps on the *b* = 0 s/mm^2^, *b* = 650 s/mm^2^ and *b* = 1000 s/mm^2^ data using a constrained non-linear least-squares algorithm.^76^ FA measures were calculated but later excluded from further analysis as FA is a non-specific measure of tissue microstructure, particularly in regions of crossing fibres.^77^

Using the neurite orientation density and dispersion imaging model, we calculated maps of ICVF from multi-shell diffusion data across all acquired *b*-values as implemented in the AMICO toolbox.^78^ We then used the previously calculated tissue-specific (GM, WM and cerebrospinal fluid) response functions and the msmt_5tt algorithm followed by the estimation of a group-average response function to calculate the fibre orientation distribution maps based on the multi-shell multi-tissue constrained spherical deconvolution method,^79^ intensity normalization of fibre orientation distributions and fibre orientation distribution peak extraction for tract segmentation.^80,81^ For tract segmentation, we used the fibre orientation distribution peaks obtained with Mrtrix3 applying the pretrained TractSeg model^82^ as described previously.^58^ The averaged MD, ICVF, R1, MTsat and R2* values were sampled within individual tracts in participants’ native space. We used the number of voxels in each tract as a proxy for its volume. For the statistical comparisons, we standardized all tract-specific values by setting them to a mean of 0 and a SD of 1. Finally, for each tract, we defined seed and target regions in the GM using SPM12’s factorization-based labelling that provided the cortical and subcortical regions for the calculation of volume and microstructure regional averages (Supplementary Fig. 1).

### Statistical analyses

Prior to our main analyses, we used the Shapiro–Wilk test to assess normality assumptions and the Levene’s test for homogeneity of variances. We selected parametric or non-parametric tests based on the distributional properties of the data. For non-parametric data, we used the Mann–Whitney U-test for comparisons between independent groups and the Welch test for paired comparisons when estimating the effect of pulsatile GnRH therapy within the DS group.

In the cross-sectional baseline analysis, we tested for group differences between DS individuals and healthy controls, across all cortical and subcortical regional averages of GM volume, MTsat, R1 and R2*. Identically, we used the same statistical design and two-tailed *t*-tests to look for differential WM tract averages of tract volume, MTsat, R1, R2*, MD and ICVF.

For the longitudinal analysis, we first calculated parameter difference maps across all GM regions and WM tracts by subtracting region by region the second from the first time point data. We then tested for between-group (DS versus healthy controls) differences using two-tailed *t*-tests. We performed *post hoc* within group tests between time points using the corresponding baseline and follow-up regional metrics in DS individuals only. The relationships between MRI indices and cognitive outcome were tested in a linear regression model.

For each of the models tested, we reported both the statistical significance (*P*-value) and the effect size (Cohen’s *d*). Significant results were presented after family-wise error (FWE) correction for multiple comparisons at *P*_FWE_  *<* 0.05, and trends were reported uncorrected for multiple comparisons at *P*  *<* 0.001. All analyses were performed using Python 3.10.12 packages NumPy 1.23.5, pandas 1.5.3, seaborn 0.12.2, sklearn 1.2.2, matplotlib 3.7.1 and statsmodels 0.14.0.

## Results

### Participants

There were no significant age or sex differences between individuals with DS and healthy controls (Table 1).

### Baseline differences: global brain metrics

**Figure 1:**
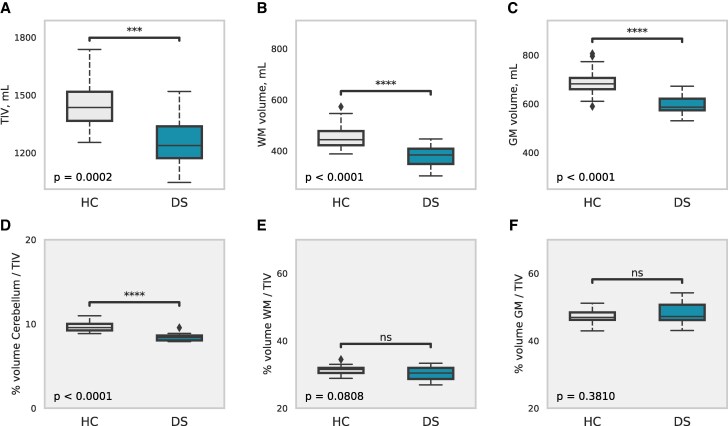
**Global brain metrics in Down syndrome (*n*  *=* 11, DS) compared with neurotypically developed healthy controlss (*n*  *=* 27, HC).** (**A–C**) Boxplots representing TIV, WM and GM volume. (**D–F**) Boxplots representing relative differences (in %) between DS and HC in cerebellum, WM and GM, after adjusting for TIV. Significance levels of the two-tailed *t*-tests denoted as ***P  *<* 0.001, ****P  *<* 0.0001 and ns, not significant. DS, Down syndrome; HC, healthy controls; GM, grey matter; TIV, total intracranial volume; WM, white matter.

Individuals with DS showed reduced global GM and WM volumes and TIV compared with healthy controls (*P*  *<* 0.001; Fig. 1A–C). After adjustment for TIV, the between-group differences in GM and WM volumes lost significance (Fig. 1D–F). Only the cerebellar volume differences between DS individuals and controls survived the TIV adjustment (*P*  *<* 0.0001; Fig. 1D). Compared with controls, the DS group showed a globally reduced MTsat in the GM (Cohen’s *d* −0.60, *P*  *=* 0.009), paralleled by higher MD (*d* 3.29, *P*  *<* 0.001) and lower ICVF (*d* −7.38, *P*  *<* 0.001) in WM.

### Baseline differences between DS individuals and healthy controls: regional brain metrics

#### Grey matter

**Figure 2:**
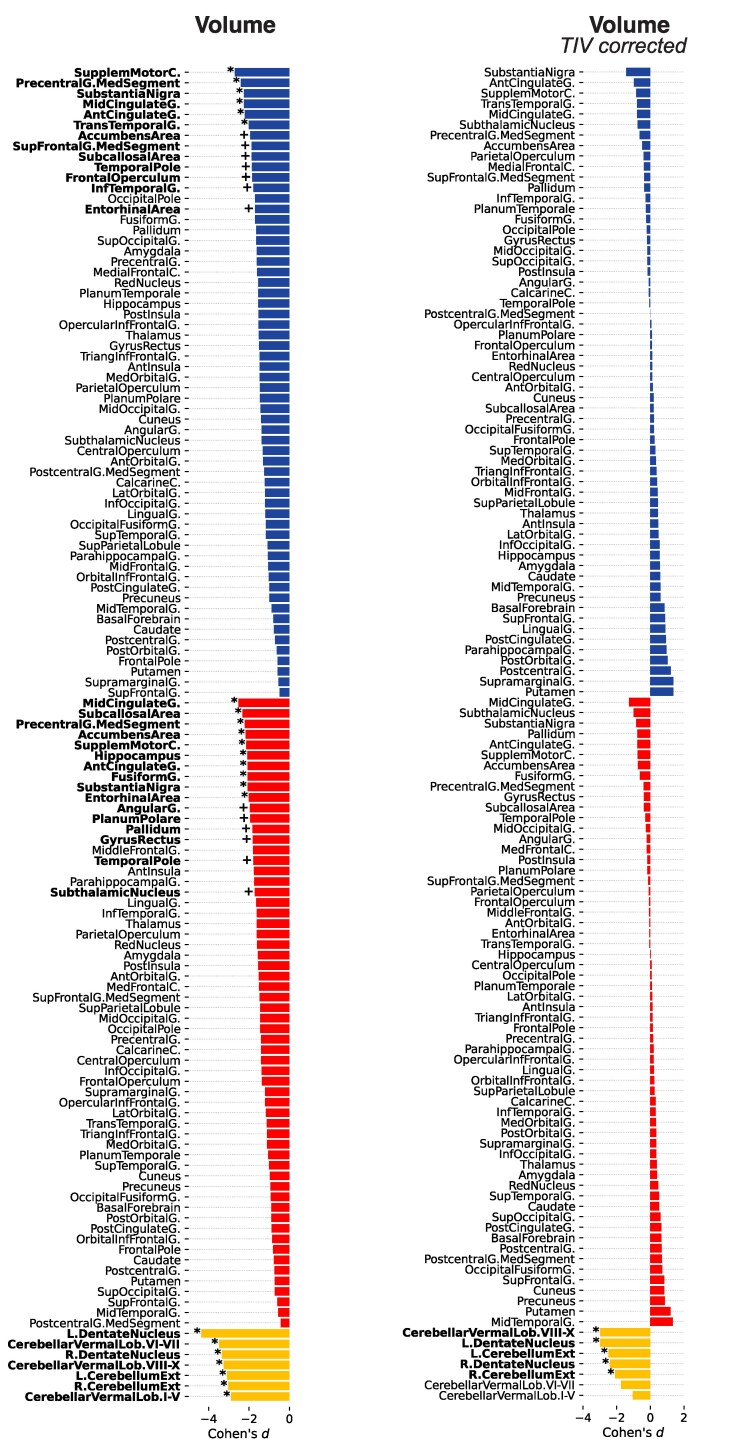
**Cross-sectional baseline comparison of regional GM volume between individuals with DS (*n*  *=* 11) and neurotypically developed controls (*n*  *=* 27).** Cohen’s *d* effect size (*y*-axis, upper third: left hemisphere; middle third: right hemisphere; lower third: cerebellar regions). *Left*: regional GM volume differences without (volume). *Right*: with TIV adjustment (volume *TIV corrected*). Significance levels of the two-tailed *t*-tests denoted with asterisk (*) *FWE-corrected P*_FWE_ < 0.05 and plus sign (+) *for uncorrected P*_uncorr_ < 0.001. DS, Down syndrome; FWE, family-wise error; GM, grey matter; TIV, total intracranial volume.

In the cross-sectional baseline comparison without correction for individuals’ TIV, we observed in DS individuals, compared with controls, a nearly symmetrical pattern of lower GM volume in the bilateral supplementary motor cortex, the medial segments of the precentral gyrus, the anterior and middle cingulate cortex, the substantia nigra, the left transtemporal gyrus and the right hemispheric subcallosal area, nucleus accumbens, fusiform gyrus, entorhinal cortex and hippocampus (*P*_FWE_ < 0.05; Fig. 2). There were trends for lower GM volume in the left hemispheric nucleus accumbens, subcallosal area, superior frontal gyrus, temporal pole, frontal operculum, inferior temporal gyrus and entorhinal cortex like the right angular gyrus, planum polare, pallidum, gyrus rectus, temporal pole and subthalamic nucleus (*P*_uncorr_ < 0.001; Fig. 2). In the TIV-adjusted cross-sectional analysis, only the cerebellum volume decreases in DS survived the correction for multiple comparisons (*P*_FWE_ < 0.05; Fig. 2).

**Figure 3:**
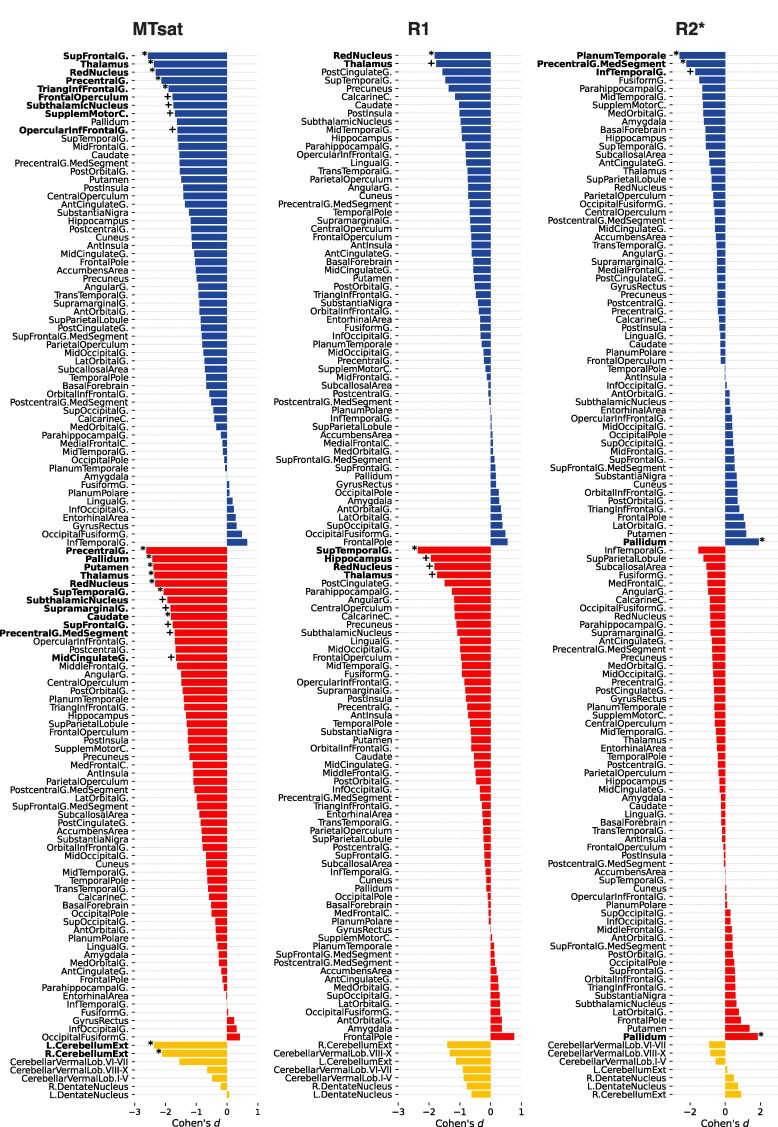
**Cross-sectional baseline comparison of regional tissue microstructure between individuals with DS (*n*  *=* 11) and neurotypically developed controls (*n*  *=* 27).** Cohen’s *d* effect size (*y*-axis, upper third: left hemisphere; middle third: right hemisphere; lower third: cerebellar regions). Regional GM microstructural MTsat, R1 and R2* differences. Significance levels of the two-tailed *t*-tests denoted with asterisk (*) *FWE-corrected P*_FWE_  *<* 0.05 and plus sign (+) *for uncorrected P*_uncorr_ < 0.001. DS, Down syndrome; FWE, family-wise error; MTsat, magnetization transfer saturation; R1, effective longitudinal relaxation rate; R2*, effective transverse relaxation rate.

The identical statistical design of the cross-sectional baseline comparison between individuals with DS and healthy controls showed lower MTsat values in DS individuals in the cerebellum, precentral gyrus, thalamus and red nucleus bilaterally, paralleled by lower MTsat in the left superior and inferior frontal gyrus, additionally to the right pallidum and striatum (*P*_FWE_ < 0.05; Fig. 3). There were between-groups trends for lower MTsat in DS bilaterally in the subthalamic nucleus, frontal and temporal cortical areas (*P*_uncorr_ < 0.001; Fig. 3), which were also largely replicated as trends in the analysis of R1 maps (*P*_uncorr_ < 0.001; Fig. 3). Compared with healthy controls, DS individuals showed higher R2* in the pallidum bilaterally (*P*_FWE_ < 0.05; Fig. 3) in addition to reduced R2* in the left planum temporale and the precentral gyrus (*P*_FWE_ < 0.05; Fig. 3). There was a trend for lower R2* in DS individuals compared with controls comprising the left inferior temporal gyrus (*P*_uncorr_ < 0.05; Fig. 3). For the detailed presentation of results, see Supplementary Table 2.

#### White matter

**Figure 4:**
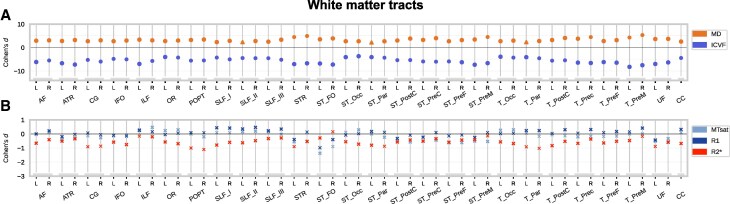
**Cross-sectional baseline comparison of tract-specific WM volume and tissue microstructure between individuals with DS (*n*  *=* 7) and neurotypically developed controls (*n*  *=* 12).** Comparison of (**A**) diffusion indices MD and ICVF and (**B**) relaxometry indices MTsat, R1 and R2* across 51 tracts. Cohen’s *d* effects sizes of the two-tailed *t*-tests denoted with solid dots (•) for *P*_FWE_ < 0.05, triangles (Δ) for *P*_uncorr_ < 0.001 and crosses (x) for non-significant results *P*_uncorr_ ≥ 0.001. DS, Down syndrome; FWE, family-wise error; ICVF, intracellular volume fraction; MD, mean diffusivity; MTsat, magnetization transfer saturation; R1, effective longitudinal relaxation rate; R2*, effective transverse relaxation rate; WM, white matter.

The cross-sectional between-group comparison at baseline showed higher MD and lower ICVF in DS participants compared with controls for all studied long association, projection and commissural WM tracts (*P*_FWE_ < 0.05; Fig. 4A). There were no significant between-group differences across WM tracts in MTsat, R1 or R2* (*P*_FWE_ < 0.05; Fig. 4B). For the detailed presentation of results, see Supplementary Table 3.

### Longitudinal differential changes in DS individuals and healthy controls: regional brain metrics

#### Grey matter

**Figure 5:**
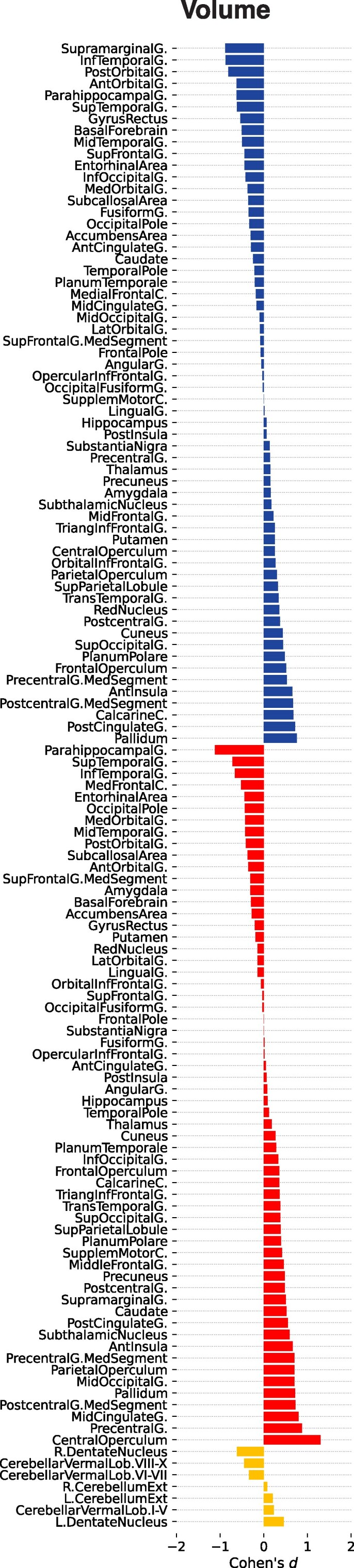
**Comparison of longitudinal changes in GM volume between individuals with DS undergoing pulsatile GnRH therapy (*n*  *=* 8) and neurotypically developed controls without intervention over 6 months (*n*  *=* 13).** Cohen’s *d* effect size (*y*-axis, upper third: left hemisphere; middle third: right hemisphere; lower third: cerebellar regions). Representation of trends in the absence of statistically significant differences between groups. DS, Down syndrome; GnRH, gonadotropin-releasing hormone; GM, grey matter.

**Figure 6:**
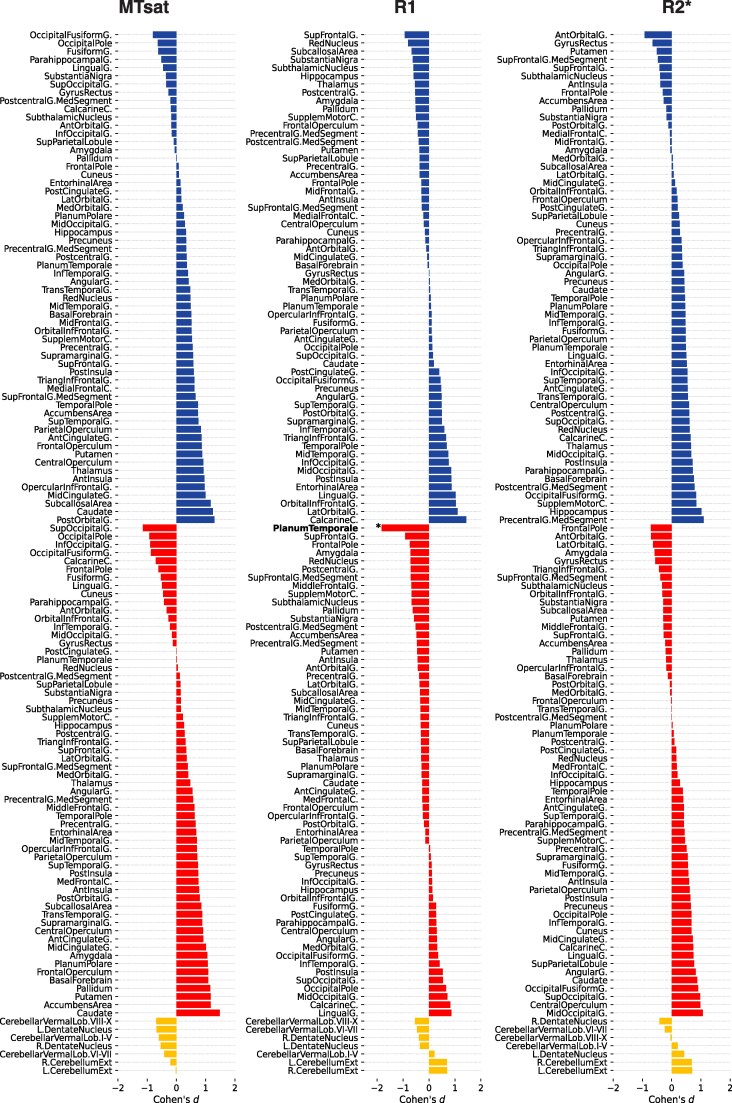
**Comparison of longitudinal changes in GM tissue microstructure between individuals with DS undergoing pulsatile GnRH therapy (*n*  *=* 8) and neurotypically developed controls without intervention over 6 months (*n*  *=* 13).** Cohen’s *d* effect size (*y*-axis, upper third: left hemisphere; middle third: right hemisphere; lower third: cerebellar regions). Regional GM microstructural MTsat, R1 and R2* differences. Significance levels of the regression analyses result on relative changes over time denoted with asterisk (*) for *FWE-corrected P*_FWE_  *<* 0.05. DS, Down syndrome; FWE, family-wise error; GnRH, gonadotropin-releasing hormone; GM, grey matter; MTsat, magnetization transfer saturation; R1, effective longitudinal relaxation rate; R2*, effective transverse relaxation rate.

In the between-group comparison, there were no differential longitudinal changes between DS individuals and healthy controls in GM volume, MTsat, R1 or R2* maps (*P*_FWE_ < 0.05; Figs 5 and 6) over the 6-month study period. We observed a trend for R1 decrease in the right planum temporale in DS individuals compared with controls (*P*_uncorr_ < 0.001; Fig. 6). Investigating regional changes beyond global longitudinal trends, and after adjustment for the corresponding global parameter differences in DS and HC,^67^ we observed higher MTsat values in the DS group in the left middle temporal gyrus and the right occipital fusiform gyrus, along with higher R2* values in the pallidum bilaterally (*P*_FWE_ < 0.05; Supplementary Fig. 2) compared with healthy controls over the 6-month period. There were trends for higher MTsat values in the left inferior temporal gyrus and lower MTsat, paralleled by a trend for lower R1 in the right thalamus in the DS group (*P*_FWE_ < 0.05; *P*_uncorr_ < 0.001; Supplementary Fig. 2).

#### White matter

**Figure 7:**
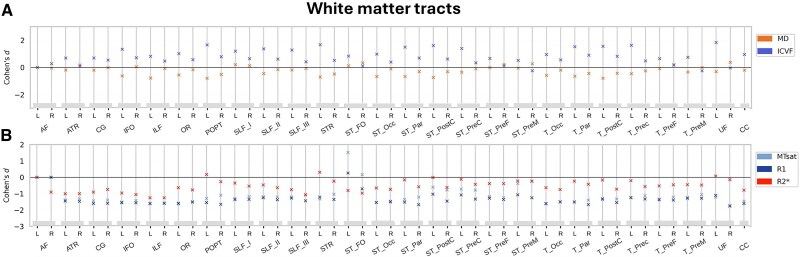
**Comparison of longitudinal changes in tract-specific WM volume and tissue microstructure between individuals with DS (*n*  *=* 7) and neurotypically developed controls (*n*  *=* 12).** (**A**) Diffusion indices MD and ICVF and (**B**) relaxometry indices MTsat, R1 and R2* across 51 tracts. Cohen’s *d* effects sizes of the regression analyses result on relative changes over time. Representation of trends in the absence of statistically significant differnces between groups, Crosses (x) denote trends at *P*_uncorr_ ≥ 0.001. DS, Down syndrome; ICVF, intracellular volume fraction; MD, mean diffusivity; MTsat, magnetization transfer saturation; R1, effective longitudinal relaxation rate; R2*, effective transverse relaxation rate; WM, white matter.

In our analysis testing for differential changes between DS individuals and healthy controls over time, there were no significant findings for MTsat, R1, R2*, MD and ICVF over the WM tracts (*P*_FWE_ < 0.05; Fig. 7A and B). For the detailed presentation of results, see Supplementary Table 4.

### Longitudinal changes of cognition and brain anatomy in DS participants

#### Cognition over time

The MoCA baseline scores in DS individuals, averaging at 11.4 ± 5.5, increased to 15.1 ± 5.6 (*P*  *<* 0.01) after 6 months of GnRH pulsatile therapy.

#### Brain anatomy over time

In the *post hoc* analysis confined to the DS group, we did not observe any significant regional changes over time neither in GM volume, MTsat, R1 or R2* (*P*_uncorr_ < 0.001) nor in WM MD, ICVF, MTsat, R1 or R2* (*P*_uncorr_ < 0.001).

#### Interaction between cognitive and brain anatomy changes over time

The *post hoc* regression analysis between MoCA improvement, regional GM volume and microstructure indices showed a trend for a negative correlation between MoCA improvement and R1 changes in cortical areas, contrasted with positive trends in bilateral prefrontal and parietal areas for MTsat and R2* (*P*_uncorr_: *<0.05, **<0.01 and ***<0.001; Supplementary Fig. 3).

The identical *post hoc* WM analyses showed a negative correlation with MTsat values in the left superior longitudinal fascicle and the thalamo-parietal projections, in addition to similar relationship with R1 in the left uncinate fascicle (*P*_FWE_ < 0.05; Supplementary Fig. 3). There were similar trends for MTsat, R1 and R2* across all major tracts.

To further explore the link between MRI metrics and cognition over time, we weighted the individual MoCA improvement by the baseline MoCA score. This revealed a negative correlation between cognitive improvement and MTsat values in the corpus callosum, the superior longitudinal fascicles, the superior thalamic radiation and thalamo-parietal projections, paralleled by negative correlations with R1 values in the left uncinate fascicle and R2* values in the anterior thalamic radiation (*P*_FWE_: *** < 0.05; Supplementary Fig. 4). In all these analyses, there were no significant GM findings. For the detailed presentation of results, see Supplementary Table 5.

## Discussion

In our longitudinal study, we showed enduring but differential brain anatomy patterns between individuals with DS undergoing GnRH pulsatile therapy and healthy controls over 6 months. Using a quantitative multi-contrast MRI strategy, we went beyond the confirmation of cerebellar volume loss and pallidum calcifications, visible even to the naked eye, to demonstrate in DS lower macromolecular content in frontal, temporal and subcortical areas, paralleled by neurite loss but preserved macromolecular/myelin content across the brain’s WM. These patterns remained stable after 6 months of GnRH therapy despite the signs of treatment-associated cognitive improvements.

Considering the controversies in the literature about brain anatomy lifespan trajectories in DS, we set out to provide a comprehensive overview on brain morphometry and tissue properties differences before studying therapy-induced longitudinal changes. Given the assumption of omnipresent Alzheimer’s disease pathology, including amyloid accumulation and neurofibrillary tangle formation above the age of 40 years,^9,11,12^ we intentionally restricted the age range of the participants to between 20 and 30 years old to minimize its confounding effects. Our main morphometry finding reveals smaller cerebellum, temporal and prefrontal cortical volumes, aligning with the most commonly reported anatomical patterns (for review, see Neale *et al*.^17^). The contrast between regional findings with and without adjustment for the DS-associated microcephaly highlights the challenge of distinguishing global effects from local ones within the context of shared disease-related variance.

The pattern of cortical and subcortical differences in MR parameters indicative of brain tissue properties represents a novel finding. The spatial overlap of MTsat and R1 results, both primarily driven by macromolecular content of myelin,^34,35^ validates our interpretation. Given the topography of myelin-related differences in cerebellar and primary motor cortical areas, we relate these findings to the overt motor control dysfunction in DS. On a similar note, the myelin reduction in the basal ganglia and thalamus, paralleled by a similar trend in cingulate, frontal, temporal and parietal areas, confirms the notion of widespread differences in the context of delayed intracortical myelination.^7,83-86^ The reported higher R2* values in the pallidum may indicate both increased iron content, as reported in the literature, and calcifications, which are frequently observed in diagnostic brain imaging of individuals with DS.^87^ Cortical and subcortical regions play a pivotal role in shaping cognitive and behavioural experiences. A post-mortem study conducted on human foetal brains affected by DS revealed a one-third reduction in neocortical cells within the frontal cortex.^88^ Together with our results, the existing evidence from the literature supports the notion of cortical volume loss predominantly related to deficient neurogenesis that remains stable over time until neurodegenerative processes—i.e. Alzheimer’s disease neuropathology prevail.

The combination of diffusion- and relaxometry-based indices of WM microstructure is a novel finding that extends previous DTI-based reports. Complementary to prior findings of tensor-based indices of FA reductions in frontal and parietal WM,^47,48^ that are biologically vaguely interpreted as differences in fibre directionality, we obtained a more complex picture showing predominant neurite loss contrasted with a relative sparing of WM’s myelin. Given the relatively young and homogenous age of our study participants and the widespread effects observed, we could not provide evidence supporting the ‘retrogenesis’ model proposing early changes in late myelinating and relative sparing of early myelinating pathways, as seen in ageing^54^ and in older individuals with DS.^51^ Though, over the short study duration, our findings aligned with the notion of diverging temporal trajectories in frontal and cerebellar cortices,^8^ with the cerebellum showing less pronounced myelin loss against a background of advanced atrophy, while frontal areas exhibited myelin reduction in frontal areas without corresponding volume decrease.

The aim of our longitudinal study was to assess GnRH therapy-induced effects. Considering the unmet challenge of recruiting a longitudinal cohort of individuals with DS not receiving GnRH therapy, we took special care to provide optimal control for time-related effects by including typically developed healthy participants also scanned at two time points 6 months apart. Along the same lines, we chose a quantitative MRI protocol that reduces the inherent time- and space-variant inhomogeneity bias of the magnetic field. Over the 6-month study period, there were no significant brain anatomy changes. This finding resonates well with the few previous longitudinal studies in older individuals with DS that reported either subtle hippocampal volume changes in the hippocampus among those with mild cognitive impairment^89^ or no changes at all after 3 years of observation.^90^ We denote the prevailing negative correlation in the *post hoc* analysis confined to the DS group between improvements in overall cognition and myelin content in specific WM tracts. Given the low level of significance, we refrain here from further speculation on the neurobiology underlying the presumed GnRH-driven processes.

Despite the unique insights gained from this study, we acknowledge several important limitations. Our analyses were conducted on a relatively modest sample size that corresponds to our already published pilot study in this population.^56^ The study lacked a control group of individuals with DS, and there was no blinding for the administered GnRH pulsatile therapy. These factors may constrain the generalizability of our findings.

In summary, our results, spanning between established morphometry and tissue microstructure quantification, offer empirical evidence supporting the potential of the proposed ‘*in vivo* histology’ strategy as foundational framework for future studies in DS. The ability to differentiate *in vivo* the contributors to the measured MR contrast not only provides straightforward neurobiological interpretation of the obtained findings but also offers a window of opportunity to discern DS prototypical neurodevelopmental anatomical features from advancing Alzheimer’s disease neuropathology. This distinction may prove increasingly important in ongoing and future therapeutic approaches using monoclonal anti-amyloid antibodies or other innovative treatments.

## Supplementary Material

fcaf117_Supplementary_Data

## Data Availability

Pre-processed and deidentified data, generated at the Laboratory for Research in Neuroimaging—LREN, University Lausanne, Switzerland, are available on request accompanied by a positive ethics vote for the intended research.
