# Supplementary material for: Enduring differential patterns of neuronal loss and myelination along 6-month pulsatile gonadotropin-releasing hormone therapy in individuals with Down syndrome
Source: Brain Commun. 2025 Mar 22;7(2):fcaf117. doi: 10.1093/braincomms/fcaf117 (PMC11969670; doi:10.1093/braincomms/fcaf117)
Supplement: fcaf117_Supplementary_Data [file fcaf117_supplementary_data.pdf]

Supplementary Material

Figures and Tables

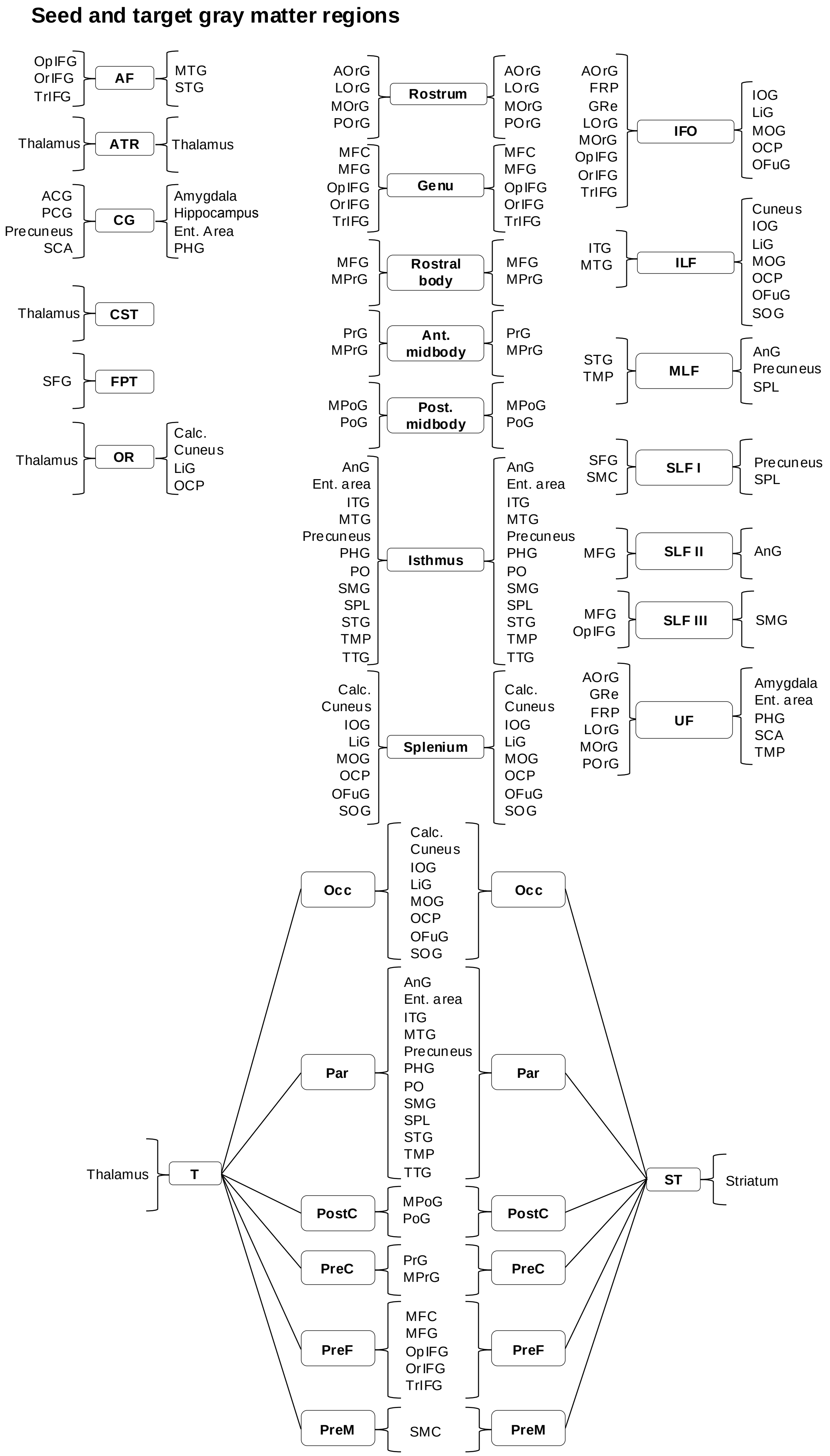

Supplementary Figure 1. Schematic representation of white matter tracts with corresponding cortical and subcortical seed and target regions for cingulate, commissural, long-association and projection fibres.

Abbreviations: ***ACG*** *- anterior cingulate gyrus;* ***AF*** *- arcuate fasciculus;* ***AnG*** *- angular gyrus;* ***AOrG*** *- anterior orbital gyrus;* ***ATR*** *- anterior thalamic radiation;* **Corpus callosum** *(rostrum, genu, rostral body, anterior midbody, posterior midbody, isthmus, splenium);* **Calc** *- calcarine cortex;* ***CG*** *- cingulum bundle;* ***CST*** *- cortico-spinal tract;* **Ent. Area** *- entorhinal area;* ***FPT*** *- fronto-pontine tract;* ***FRP*** *- frontal pole;* **GRe** *- gyrus rectus;* **Hippoc** *- hippocampus;* ***IFO*** *- inferior fronto-occipital fasciculus;* ***ILF*** *- inferior longitudinal fasciculus;* ***IOG*** *- inferior occipital gyrus;* ***ITG*** *- inferior temporal gyrus;* ***LiG*** *- lingual gyrus;* ***LOrG*** *- lateral orbital gyrus;* ***MFC*** *- medial frontal cortex;* ***MFG*** *- middle frontal gyrus;* ***MLF*** *- middle longitudinal fascicle;* ***MOG*** *- middle occipital gyrus;* ***MOrG*** *- medial orbital gyrus;* ***MPoG*** *- postcentral gyrus medial segment;* ***MPrG*** *- precentral gyrus medial segment;* ***MTG*** *- middle temporal gyrus;* ***OCP*** *- occipital pole;* ***OFuG*** *- occipital fusiform gyrus;* ***OpIFG*** *- opercular part of the inferior frontal gyrus;* ***OR*** *- optic radiation;* ***OrIFG*** *- orbital part of the inferior frontal gyrus;* ***PCG*** *- posterior cingulate gyrus;* ***PHG*** *- prahippocampal gyrus;* ***PO*** *- parietal operculum;* **PoG** *- postcentral gyrus;* ***POrG*** *- posterior orbital gyrus;* ***POPT*** *- parieto-occipital pontine tract;* **Precun** *- precuneus;* ***PrG*** *- precentral gyrus;* ***SCA*** *- subcallosal area;* ***SFG*** *- superior frontal gyrus;* ***SLF*** *- superior longitudinal fasciculus;* ***SMC*** *- supplementary motor cortex;* ***SMG*** *- supramarginal gyrus;* ***SOG*** *- superior occipital gyrus;* ***SPL*** *- superior parietal lobule;* ***STG*** *- superior temporal gyrus;* ***ST_FO*** *- striato-fronto-orbital;* ***ST_Occ*** *- striato-occipital;* ***ST_Par*** *- striato-parietal;* ***ST_PostC*** *- striato-postcentral;* ***ST_PreC*** *- striato-precentral;* ***ST_PreF*** *- striato-prefrontal;* ***ST_PreM*** *- striato-premotor;* ***TMP*** *- temporal pole;* ***T_Occ*** *- thalamo-occipital;* ***T_Par*** *- thalamo-parietal;* ***T_PostC*** *- thalamo-postcentral;* ***T_PreC*** *- thalamo-precentral;* ***T_PreF*** *- thalamo-prefrontal;* ***T_PreM*** *- thalamo-premotor;* ***TrIFG*** *- triangular part of the inferior frontal gyrus;* ***TTG*** *- transverse temporal gyrus;* ***UF*** *- uncinate fasciculus.*

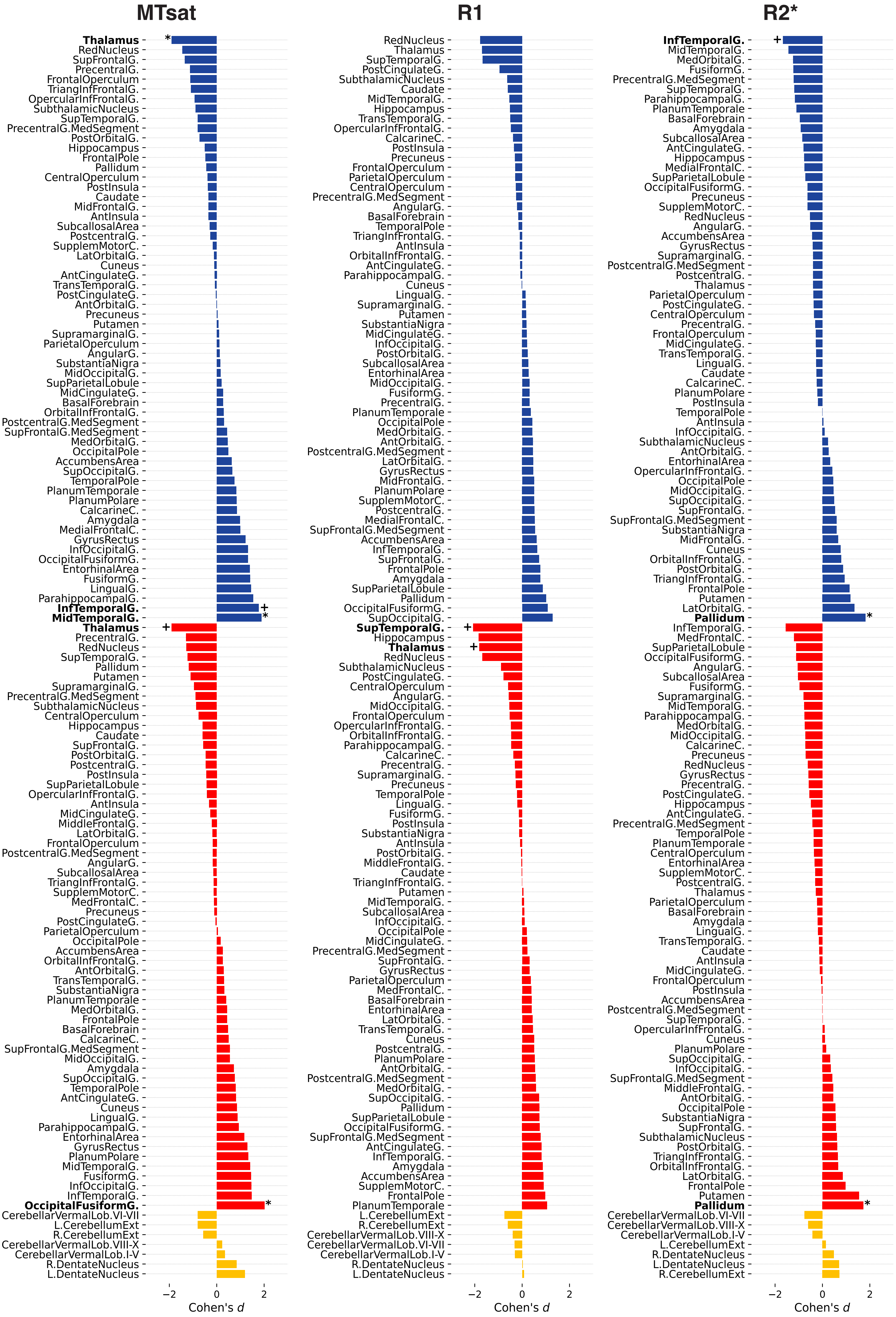
MTsat
Thalamus
RedNucleus
SupFrontalG.
PrecentralG.
FrontalOperculum
TriangInfFrontalG.
OpercularInfFrontalG.
SubthalamicNucleus
SupTemporalG.
PrecentralG.MedSegment
PostOrbitalG.
Hippocampus
FrontalPole
Pallidum
CentralOperculum
PostInsula
Caudate
MidFrontalG.
AntInsula
SubcallosalArea
PostcentralG.
SupplemMotorC.
LatOrbitalG.
Cuneus
AntCingulateG.
TransTemporalG.
PostCingulateG.
AntOrbitalG.
Precuneus
Putamen
SupramarginalG.
ParietalOperculum
AngularG.
SubstantiaNigra
MidOccipitalG.
SupParietalLobule
MidCingulateG.
BasalForebrain
OrbitalInfFrontalG.
PostcentralG.MedSegment
SupFrontalG.MedSegment
MedOrbitalG.
OccipitalPole
AccumbensArea
SupOccipitalG.
TemporalPole
PlanumTemporale
PlanumPolare
CalcarineC.
Amygdala
MedialFrontalC.
GyrusRectus
InfOccipitalG.
OccipitalFusiformG.
EntorhinalArea
FusiformG.
LingualG.
ParahippocampalG.
InfTemporalG.
MidTemporalG.
Thalamus
PrecentralG.
RedNucleus
SupTemporalG.
Pallidum
Putamen
SupramarginalG.
PrecentralG.MedSegment
SubthalamicNucleus
CentralOperculum
Hippocampus
Caudate
SupFrontalG.
PostOrbitalG.
PostcentralG.
PostInsula
SupParietalLobule
OpercularInfFrontalG.
AntInsula
MidCingulateG.
MiddleFrontalG.
LatOrbitalG.
FrontalOperculum
PostcentralG.MedSegment
AngularG.
SubcallosalArea
TriangInfFrontalG.
SupplemMotorC.
MedFrontalC.
Precuneus
PostCingulateG.
ParietalOperculum
OccipitalPole
AccumbensArea
OrbitalInfFrontalG.
AntOrbitalG.
TransTemporalG.
SubstantiaNigra
PlanumTemporale
MedOrbitalG.
FrontalPole
BasalForebrain
CalcarineC.
SupFrontalG.MedSegment
MidOccipitalG.
Amygdala
SupOccipitalG.
TemporalPole
AntCingulateG.
Cuneus
LingualG.
ParahippocampalG.
EntorhinalArea
GyrusRectus
PlanumPolare
MidTemporalG.
FusiformG.
InfOccipitalG.
InfTemporalG.
OccipitalFusiformG.
CerebellarVermalLob.VI-VII
L.CerebellumExt
R.CerebellumExt
CerebellarVermalLob.VIII-X
CerebellarVermalLob.I-V
R.DentateNucleus
L.DentateNucleus
−2 0 2
Cohen's d
R1
RedNucleus
Thalamus
SupTemporalG.
PostCingulateG.
SubthalamicNucleus
Caudate
MidTemporalG.
Hippocampus
TransTemporalG.
OpercularInfFrontalG.
CalcarineC.
PostInsula
Precuneus
FrontalOperculum
ParietalOperculum
CentralOperculum
PrecentralG.MedSegment
AngularG.
BasalForebrain
TemporalPole
TriangInfFrontalG.
AntInsula
OrbitalInfFrontalG.
AntCingulateG.
ParahippocampalG.
Cuneus
LingualG.
SupramarginalG.
Putamen
SubstantiaNigra
MidCingulateG.
InfOccipitalG.
PostOrbitalG.
SubcallosalArea
EntorhinalArea
MidOccipitalG.
FusiformG.
PrecentralG.
PlanumTemporale
OccipitalPole
MedOrbitalG.
AntOrbitalG.
PostcentralG.MedSegment
LatOrbitalG.
GyrusRectus
MidFrontalG.
PlanumPolare
SupplemMotorC.
PostcentralG.
MedialFrontalC.
SupFrontalG.MedSegment
AccumbensArea
InfTemporalG.
SupFrontalG.
FrontalPole
Amygdala
SupParietalLobule
Pallidum
OccipitalFusiformG.
SupOccipitalG.
SupTemporalG.
Hippocampus
Thalamus
RedNucleus
SubthalamicNucleus
PostCingulateG.
CentralOperculum
AngularG.
MidOccipitalG.
FrontalOperculum
OpercularInfFrontalG.
OrbitalInfFrontalG.
ParahippocampalG.
CalcarineC.
PrecentralG.
SupramarginalG.
Precuneus
TemporalPole
LingualG.
FusiformG.
PostInsula
SubstantiaNigra
AntInsula
PostOrbitalG.
MiddleFrontalG.
Caudate
TriangInfFrontalG.
Putamen
MidTemporalG.
SubcallosalArea
InfOccipitalG.
OccipitalPole
MidCingulateG.
PrecentralG.MedSegment
SupFrontalG.
GyrusRectus
ParietalOperculum
MedFrontalC.
BasalForebrain
EntorhinalArea
LatOrbitalG.
TransTemporalG.
Cuneus
PostcentralG.
PlanumPolare
AntOrbitalG.
PostcentralG.MedSegment
MedOrbitalG.
SupOccipitalG.
Pallidum
SupParietalLobule
OccipitalFusiformG.
SupFrontalG.MedSegment
AntCingulateG.
InfTemporalG.
Amygdala
AccumbensArea
SupplemMotorC.
FrontalPole
PlanumTemporale
L.CerebellumExt
R.CerebellumExt
CerebellarVermalLob.VIII-X
CerebellarVermalLob.VI-VII
CerebellarVermalLob.I-V
R.DentateNucleus
L.DentateNucleus
−2 0 2
Cohen's d
R2*
InfTemporalG.
MidTemporalG.
MedOrbitalG.
FusiformG.
PrecentralG.MedSegment
SupTemporalG.
ParahippocampalG.
PlanumTemporale
BasalForebrain
Amygdala
SubcallosalArea
AntCingulateG.
Hippocampus
MedialFrontalC.
SupParietalLobule
OccipitalFusiformG.
Precuneus
SupplemMotorC.
RedNucleus
AngularG.
AccumbensArea
GyrusRectus
SupramarginalG.
PostcentralG.MedSegment
PostcentralG.
Thalamus
ParietalOperculum
PostCingulateG.
CentralOperculum
PrecentralG.
FrontalOperculum
MidCingulateG.
TransTemporalG.
LingualG.
Caudate
CalcarineC.
PlanumPolare
PostInsula
TemporalPole
AntInsula
InfOccipitalG.
SubthalamicNucleus
AntOrbitalG.
EntorhinalArea
OpercularInfFrontalG.
OccipitalPole
MidOccipitalG.
SupOccipitalG.
SupFrontalG.
SupFrontalG.MedSegment
SubstantiaNigra
MidFrontalG.
Cuneus
OrbitalInfFrontalG.
PostOrbitalG.
TriangInfFrontalG.
FrontalPole
Putamen
LatOrbitalG.
Pallidum
InfTemporalG.
MedFrontalC.
SupParietalLobule
OccipitalFusiformG.
AngularG.
SubcallosalArea
FusiformG.
SupramarginalG.
MidTemporalG.
ParahippocampalG.
MedOrbitalG.
MidOccipitalG.
CalcarineC.
Precuneus
RedNucleus
GyrusRectus
PrecentralG.
PostCingulateG.
Hippocampus
AntCingulateG.
PrecentralG.MedSegment
TemporalPole
PlanumTemporale
CentralOperculum
EntorhinalArea
SupplemMotorC.
PostcentralG.
Thalamus
ParietalOperculum
BasalForebrain
Amygdala
LingualG.
TransTemporalG.
Caudate
AntInsula
MidCingulateG.
FrontalOperculum
PostInsula
AccumbensArea
PostcentralG.MedSegment
SupTemporalG.
OpercularInfFrontalG.
Cuneus
PlanumPolare
SupOccipitalG.
InfOccipitalG.
SupFrontalG.MedSegment
MiddleFrontalG.
AntOrbitalG.
OccipitalPole
SubstantiaNigra
SupFrontalG.
SubthalamicNucleus
PostOrbitalG.
TriangInfFrontalG.
OrbitalInfFrontalG.
LatOrbitalG.
FrontalPole
Putamen
Pallidum
CerebellarVermalLob.VI-VII
CerebellarVermalLob.VIII-X
CerebellarVermalLob.I-V
L.CerebellumExt
R.DentateNucleus
L.DentateNucleus
R.CerebellumExt
−2 0 2
Cohen's d

Supplementary Figure 2. Cross-sectional baseline comparison of regional grey matter MTsat, R1 and R2* between individuals with Down syndrome (DS, n = 11) **and neurotypically developed controls** (HC, n = 27) **adjusted for the corresponding global parameter differences**. Cohen's *d* effect size (y-axis, **blue**: left hemisphere; **red**: right hemisphere; **yellow**: cerebellar regions). Significance levels of the two-tailed t-tests denoted with asterisk (*) *FWE-corrected* $p_{FWE}<.05$; plus sign (+) *for uncorrected* $p_{uncorr}<.001$.

Abbreviations: MTsat - magnetization transfer saturation; R1- effective longitudinal relaxation rate; R2 - effective transverse relaxation rate*

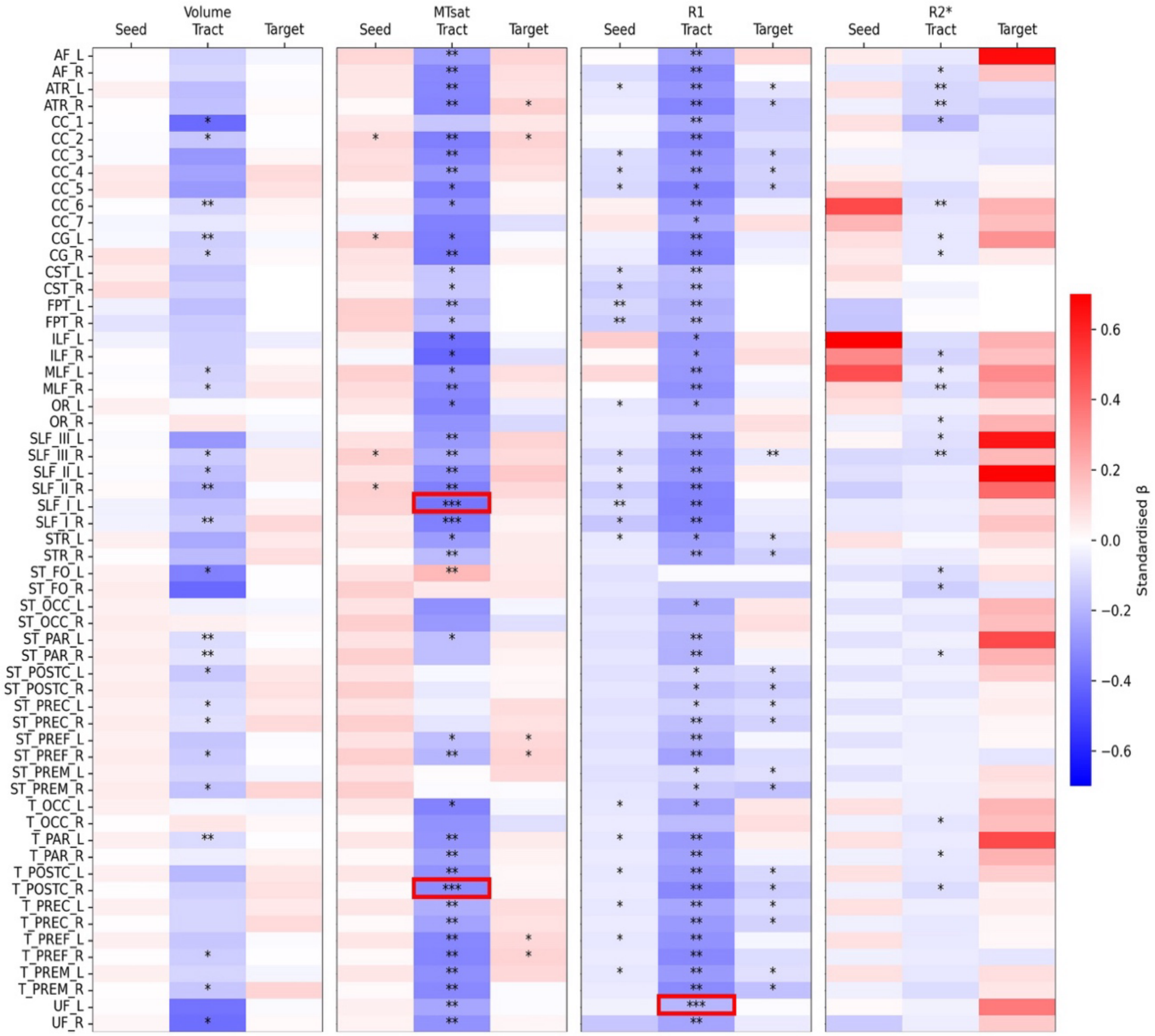

Supplementary Figure 3. Post hoc longitudinal analysis of brain anatomy and cognition changes confined to the Down syndrome group (n = 7). Combined representation of associations between changes in overall cognitive performance according to the Montreal Cognitive Assessment (MoCA) and volume, MTsat, R1, R2* differences across white matter tracts and corresponding cortical and subcortical grey matter seed and target regions with and representation of results. **Red** - positive, **blue** - negative associations. Significance levels of the regression analyses denoted with asterisk (* p<.05, ** p<.01, *** p<.001 all uncorrected for multiple comparisons), red rectangle - FWE-corrected p_{FWE}<.05).

*Abbreviations: **AF** - arcuate fasciculus; **ATR** - anterior thalamic radiation; **CC** - corpus callosum (1 = rostrum, 2 = genu, 3 = rostral body, 4 = anterior midbody, 5 = posterior midbody, 6 = isthmus, 7 = splenium); **CG** - cingulum bundle; **CST** - cortico-spinal tract; **FPT** - fronto-pontine tract; **IFO** -*

inferior fronto-occipital fasciculus; ***ILF*** *- inferior longitudinal fasciculus;* ***MLF*** *- middle longitudinal fascicle;* ***OR*** *- optic radiation;* ***POPT*** *- parieto-occipital pontine tract;* ***SLF*** *- superior longitudinal fasciculus;* ***STR*** *- superior thalamic radiation;* ***ST_FO*** *- striato-fronto-orbital;* ***ST_Occ*** *- striato-occipital;* ***ST_Par*** *- striato-parietal;* ***ST_PostC*** *- striato-postcentral;* ***ST_PreC*** *- striato-precentral;* ***ST_PreF*** *- striato-prefrontal;* ***ST_PreM*** *- striato-premotor;* ***T_Occ*** *- thalamo-occipital;* ***T_Par*** *- thalamo-parietal****; T_PostC*** *- thalamo-postcentral;* ***T_PreC*** *- thalamo-precentral;* ***T_PreF*** *- thalamo-prefrontal****; T_PreM*** *- thalamo-premotor;* ***UF*** *- uncinate fasciculus.*

MTsat - magnetization transfer saturation; R1- effective longitudinal relaxation rate; R2 - effective transverse relaxation rate.*

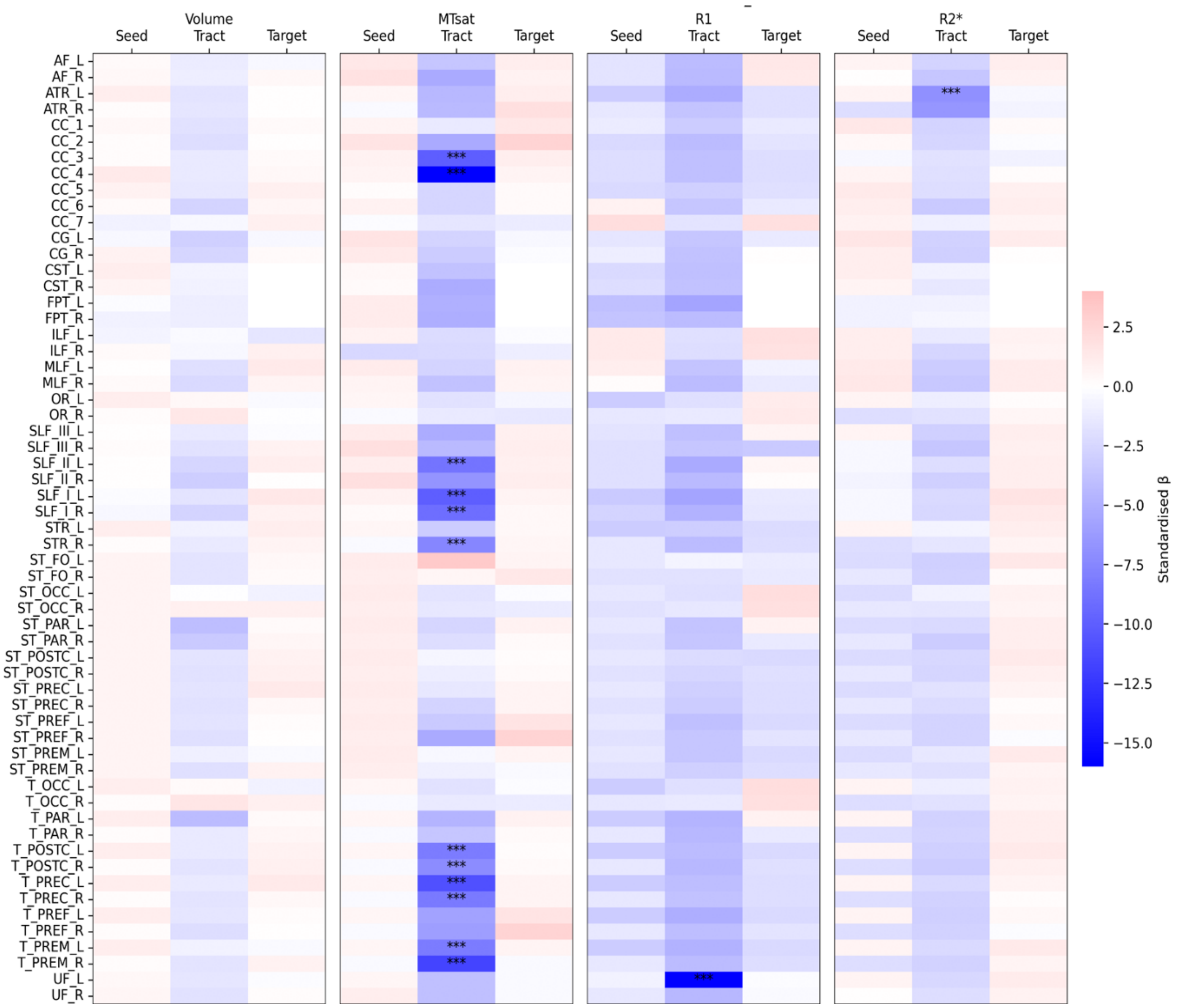

Supplementary Figure 4. Post hoc longitudinal analysis of brain anatomy and cognition changes weighted by initial cognitive performance confined to the Down syndrome group (n = 7). Combined representation of associations between changes in weighted overall cognitive performance according to the Montreal Cognitive Assessment (MoCA) and volume, MTsat, R1, R2* differences across white matter tracts and corresponding cortical and subcortical grey matter seed and target regions with and representation of results. **Red** - positive, **blue** - negative associations. Significance levels of the regression analyses denoted with asterisk (* p<.05, ** p<.01, *** p<.001 all uncorrected for multiple comparisons), red rectangle - FWE-corrected p_{FWE}<.05).

*Abbreviations: **AF** - arcuate fasciculus; **ATR** - anterior thalamic radiation; **CC** - corpus callosum (1 = rostrum, 2 = genu, 3 = rostral body, 4 = anterior midbody, 5 = posterior midbody, 6 = isthmus, 7 = splenium); **CG** - cingulum bundle; **CST** - cortico-spinal tract; **FPT** - fronto-pontine tract; **IFO** - inferior fronto-occipital fasciculus; **ILF** - inferior longitudinal fasciculus; **MLF** - middle longitudinal*

fascicle; ***OR*** *- optic radiation;* ***POPT*** *- parieto-occipital pontine tract;* ***SLF*** *- superior longitudinal fasciculus;* ***STR*** *- superior thalamic radiation;* ***ST_FO*** *- striato-fronto-orbital;* ***ST_Occ*** *- striato-occipital;* ***ST_Par*** *- striato-parietal;* ***ST_PostC*** *- striato-postcentral;* ***ST_PreC*** *- striato-precentral;* ***ST_PreF*** *- striato-prefrontal;* ***ST_PreM*** *- striato-premotor;* ***T_Occ*** *- thalamo-occipital;* ***T_Par*** *- thalamo-parietal****; T_PostC*** *- thalamo-postcentral;* ***T_PreC*** *- thalamo-precentral;* ***T_PreF*** *- thalamo-prefrontal****; T_PreM*** *- thalamo-premotor;* ***UF*** *- uncinate fasciculus.*

MTsat - magnetization transfer saturation; R1- effective longitudinal relaxation rate; R2 - effective transverse relaxation rate.*

Study	Participants/ dementia	Age range (years)	Structural analysis	Regions with lower GM in DS	Regions with increased GM in DS
**Children to young adults with Down syndrome**					
Jernigan & Bellugi (1990)	3 DS<br>14 TDC	DS (14-17)<br>TDC (8-32)	ROI analysis using manual volumetry	Whole brain, cerebellum, vermal lobules (I-V)	None reported
Jernigan *et al.* (1993)	6 DS<br>21 TDC	10-20	ROI analysis using manual volumetry	Cerebellum, caudate, frontal cortex, limbic structures (uncus, amygdala, hippocampus, parahippocampal gyrus)	None reported
Pinter *et al.* (2001)	16/0 DS<br>15 TDC	5–23	ROI analysis using manual volumetry	Whole brain, cerebellum	Parietal lobe, basal ganglia (incl. thalamus)
Kates *et al.* (2002)	12 DS<br>8 TDC	3-8	ROI analysis using manual volumetry	parietal and temporal lobes	None reported
Kaufmann *et al.* 2003	11 DS<br>22 TD	3-9	ROI analysis using manual volumetry	Posterior vermi (lobules VI-VII and VIII-X)	None reported
Carter *et al.* (2008)	30 DS<br>22 TDC	3-15	ROI analysis using manual volumetry	Whole brain, frontal and parietal lobe, cerebellum	None reported
Menghini *et al.* (2011)	12 DS<br>12 TDC	12–19	VBM using SPM2	Whole brain, L posterior cerebellum, R inferior temporal gyrus, fusiform gyrus,<br>R hippocampus	L anterior cerebellum, R fusiform gyrus, putamen, caudate, insula, superior frontal gyrus,<br>R superior and middle temporal gyrus, inferior frontal gyrus
Smigielska-Kuzia *et al.* (2011)	23/0 DS<br>26 TDC	2–15	ROI analysis using manual volumetry	Frontal lobe, temporal lobe, hippocampus, amygdala	None reported
Carducci *et al.* (2013)	21 DS<br>27 TDC	7-16	VBM using SPM2	regions of frontal lobe, cerebellum, cingulate gyrus, R middle frontal gyrus, inferior frontal gyri, L pre/postcentral gyri, hippocampus, parahippocampal gyri	Inferior parietal lobule, L superior parietal lobule, L precuneus, L middle temporal gyrus, R postcentral gyrus, R lentiform nucleus, R putamen, R claustrum

Lee *et al.* (2016)	31 DS<br>45 TDC	5-24	ROI analysis using manual volumetry	Frontal, temporal, occipital lobes	None reported
Gunbey *et al.* (2017)	10 DS<br>8 TDC	2-4	ROI analysis using FreeSurfer volumetry followed by manual check	thalamus, caudate, L putamen, L pallidum, cerebellar cortex, brainstem, corpus callosum	None reported
Fuji *et al.* (2017)	32 DS<br>32 TDC	0-11	ROI analysis using manual volumetry	brainstem	None reported
Patkee *et al.* (2020)	30+21 DS 52 +21 TDC *(fetus+neonate)*	< 1	3D reconstruction (Snapshot MRI) followed by manual volumetry	Whole brain, cerebellum	None reported
McCann *et al.* (2021)	73 DS<br>993 TDC	0-22	ROI analysis using FreeSurfer	Whole brain, planum temporale, brainstem, cerebellum, insula, cingulate gyri, putamen, parahippocampal gyri.	Perirhinal cortex, entorhinal cortex, choroid plexus, primary somatosensory cortex (Brodmann Areas 3a and 3b)
Hamadelseed & Skutella (2021)	13 DS<br>12 TDC	4-25	ROI analysis ısing volBrain	Cerebrum, cerebellum, brainstem, hippocampus, parahippocampal gyrus, frontal / parietal / temporal lobes, fusiform / superior temporal / supramarginal / angular gyri	None reported
**Non–demented adults with Down syndrome**					
Weis *et al.* (1991)	7/0 DS<br>7 TDC	30–45	ROI analysis using manual volumetry	Whole brain, cerebellum	None reported
Kesslak *et al.* (1994)	13/0 DS<br>10 TDC	23–51	ROI analysis using manual volumetry	Frontal cortex, cerebellum, hippocampus	Parahippocampal gyrus

Raz *et al.* (1995)	13/0 DS<br>12 TDC	22–50	ROI analysis using manual volumetry	Whole brain, cerebellum, hippocampus	Parahippocampal gyrus
Roth *et al.* (1996)	30/10 DS<br>30 TDC	23–60	Visual scoring for atrophy	Basal ganglia	None reported
Frangou *et al.* (1997)	17/4 DS<br>17 TDC	30–60	ROI analysis using manual volumetry	Whole brain, planum temporale	None reported
Aylward *et al.* (1997a)	30/5 DS<br>30 TDC	25–63	ROI analysis using manual volumetry	Whole brain, cerebellum	None reported
Aylward *et al.* (1997b)	22/0 DS<br>22 TDC	25–60	ROI analysis using manual volumetry	Whole brain	Putamen
Pearlson *et al.* (1998)	50/11 DS<br>23 TDC	30-55	ROI analysis using manual volumetry	Whole brain, hippocampus, amygdala	None reported
Aylward *et al.* (1999)	25/8 DS<br>25 TDC	26–59	ROI analysis using manual volumetry	Hippocampus, amygdala	None reported
Krasuski *et al.* (2002)	34/0 DS<br>33 TDC	25–64	ROI analysis using manual volumetry	Amygdala, hippocampus, posterior parahippocampal gyrus	None reported
Prasher *et al.* (2003)	24/11 DS<br>0 TDC	26–78	ROI analysis using manual volumetry	Some evidence for temporal lobe	None reported
Teipel *et al.* (2004)	34/0 DS<br>31 TDC	25–64	ROI analysis using manual volumetry	Whole brain, hippocampus	None reported
White *et al.* (2003)	19/0 DS<br>11 TDC	34–56	VBM using SPM99	Whole brain, cerebellum, L medial frontal lobe,<br>R superior/middle temporal lobe, cingulate gyrus, L hippocampus	Brainstem, L parahippocampal gyrus
Beacher *et al.* (2009)	58/19 DS<br>0 TDC	16–66	ROI analysis using manual volumetry	Whole brain, hippocampus, R amygdala, caudate, putamen	None reported

Beacher *et al.* (2010)	39/0 DS 42 TDC	18–66	ROI analysis using manual volumetry	Whole brain, L frontal lobe, cerebellum	Parietal lobe, putamen, occipital lobe
Mullins *et al.* (2013)	64/19 DS 43 TDC	22-80	ROI analysis using manual volumetry	Whole brain, hippocampus	None reported
Matthews *et al.* (2016)	12/0 DS 12 TDC	DS (32-61) TDC (60-65)	multivariate machine learning	Cerebellum occipital cortex, hippocampus, cingulate, temporal cortex	Putamen, inferior parietal cortex
Annus *et al.* (2017)	46/9 DS 30 TDC	28-65	ROI defined in MNI space (SPM)	Hippocampus	putamen
Lao *et al.* (2017)	52 DS	30-50	ROI defined in MNI space (SPM)	No significant change in GM volume in any of the investigated ROIs.	
Bletsch *et al.* (2018)	26/0 DS 23 TDC	18-51	Cortical reconstruction (FreeSurfer)	Whole brain, temporal lobe, cingulate cortices, L frontal lobe, R parietal lobe	None reported
Koenig *et al.* (2021)	34 DS 27 TDC	15-36	ROI analysis using manual volumetry	Hippocampus	None reported

Supplementary Table 1. Overview of previously published volumetry and morphometry studies in adults with Down syndrome. Individuals with Down syndrome diagnosed with dementia are denoted by the second value.

Abbreviations: DS – individuals with DS; TDC – typically developing control participants; GM – grey matter; L – left; R – right; MNI - Montreal Neurosciences Institute; ROI – region–of–interest; VBM – voxel–based–morphometry; SPM99/2 – Statistical Parametric Mapping neuroimaging analysis software, versions 99 and 2, respectively.

**Volume**		side	Cohen's *d*	p FWE
**DS < CTR**	*Accumbens Area*	R	-2.16	0.018
	Ant Cingulate G	L	-2.21	0.020
		R	-2.07	0.049
	Entorhinal Area	R	-2.01	0.044
	Fusiform G	R	-2.07	0.021
	Hippocampus	R	-2.08	0.021
	Middle Cingulate G	L	-2.25	0.013
		R	-2.52	0.003
	Precentral G Medial Segment	L	-2.41	0.006
		R	-2.21	0.031
	Subcallosal Area	R	-2.32	0.012
	Substantia Nigra	L	-2.26	0.013
		R	-2.07	0.028
	Supplementary Motor Cortex	L	-2.70	0.002
		R	-2.14	0.026
	Transverse Temporal G	L	-2.01	0.032
	Cerebellar Vermal Lobules I - V	-	-2.89	0.001
	Cerebellar Vermal Lobules VI - VII	-	-3.49	0.0001
	Cerebellar Vermal Lobules VIII - X	-	-3.26	0.0002
	Cerebellum Exterior	L	-3.09	0.0004
		R	-3.03	0.001
	Dentate Nucleus	L	-4.36	<0.0001
		R	-3.34	0.0002
**Volume** (adjusted by TIV)		**side**	**Cohen's *d***	**p FWE**
	Cerebellar Vermal Lobules VIII - X	-	-2.98	0.0001
	Cerebellum Ext.	L	-2.48	0.003
		R	-2.09	0.016
	Dentate Nucleus	L	-2.97	0.0001
		R	-2.41	0.005
**Magnetization transfer saturation (MTsat)**		**side**	**Cohen's *d***	**p FWE**
**DS < CTR**	*Caudate*	R	-1.82	0.040
	Pallidum	R	-2.43	0.001
	Precentral G	L	-2.13	0.009
		R	-2.62	0.001
	Putamen	R	-2.39	0.002
	Red Nucleus	L	-2.32	0.007
		R	-2.33	0.010
	Sup Frontal G	L	-2.57	0.001
	Sup Temporal G	R	-2.06	0.016
	Thalamus	L	-2.37	0.002
		R	-2.36	0.005
	Triang part of the Inf Frontal G	L	-1.90	0.023
	Cerebellum Exterior	L	-2.37	0.010
		R	-2.11	0.035
**Effective longitudinal relaxation time (R1)**		**side**	**Cohen's *d***	**p FWE**
**DS < CTR**	*Sup Temporal G*	R	-2.36	0.006
	Red Nucleus	L	-1.82	0.049
**Effective transverse relaxation time (R2*)**		**side**	**Cohen's *d***	**p FWE**
**DS < CTR**	*Planum Temporale*	L	-2.60	0.004
	Precentral G Medial Segment	L	-2.21	0.011

**DS > CTR**	*Pallidum*	L	1.91	0.017
		R	1.84	0.024

Supplementary Table 2. Cross-sectional study: differences in grey matter regions between individuals with Down syndrome and healthy controls.

		**Mean diffusivity (MD)**		**Intra-cellular volume fraction (ICVF)**	
**Tract**	**side**	**Cohen's *d***	**p FWE**	**Cohen's *d***	**p FWE**
Arcuate fasciculus (AF)	L	2.91	0.004	-6.16	0.006
	R	3.07	0.002	-5.50	<0.0001
Anterior thalamic radiation (ATR)	L	2.80	0.006	-6.68	<0.0001
	R	3.21	0.001	-7.25	<0.0001
Corpus callosum (CC)	-	2.56	0.011	-4.43	<0.0001
Cingulate bundle (CG)	L	2.72	0.007	-5.24	<0.0001
	R	3.07	0.001	-5.97	<0.0001
Inferior fronto-occipital fasciculus (IFO)	L	2.72	0.008	-4.81	<0.0001
	R	3.00	0.003	-5.02	<0.0001
Inferior longitudinal fasciculus (ILF)	L	3.38	0.001	-6.96	<0.0001
	R	3.07	0.002	-5.70	<0.0001
Optic radiation (OR)	L	2.76	0.007	-4.02	0.0001
	R	2.99	0.003	-4.26	<0.0001
Parieto-occipital pontine (POPT)	L	3.23	0.001	-5.56	<0.0001
	R	3.44	0.000	-5.49	<0.0001
Superior longitudinal fasciculus I (SLF_I)	L	2.34	0.023	-4.26	0.0001
	R	2.86	0.003	-5.02	<0.0001
Superior longitudinal fasciculus II (SLF_II)	L	2.30	0.034	-4.46	<0.0001
	R	2.78	0.005	-4.52	<0.0001
Superior longitudinal fasciculus III (SLF_III)	L	2.50	0.019	-4.53	<0.0001
	R	3.34	0.001	-5.18	<0.0001
Striato-fronto-orbital (ST_FO)	L	3.52	0.0005	-6.80	<0.0001
	R	3.89	0.0001	-7.23	<0.0001
Striato-occipital (ST_OCC)	L	2.70	0.008	-4.08	0.0001
	R	2.69	0.010	-3.67	0.0003
Striato-parietal (ST_PAR)	L	2.16	0.070	-4.07	0.0001
	R	2.68	0.007	-4.31	<0.0001
Striato-postcentral (ST_POSTC)	L	3.02	0.003	-5.36	<0.0001
	R	3.84	0.0001	-5.33	<0.0001
Striato-precentral (ST_PREC)	L	3.26	0.001	-5.92	<0.0001
	R	4.00	0.0001	-6.01	<0.0001
Striato-prefrontal (ST_PREF)	L	2.70	0.009	-5.91	0.006
	R	3.19	0.001	-6.25	<0.0001

Striato-premotor (ST_PREM)	L	3.45	0.0004	-7.31	<0.0001
	R	4.51	<0.0001	-6.63	<0.0001
Superior thalamic radiation (STR)	L	4.48	<0.0001	-7.05	<0.0001
	R	4.93	<0.0001	-6.70	0.006
Thalamo-occipital (T_OCC)	L	2.72	0.008	-3.91	0.0002
	R	2.97	0.003	-4.28	<0.0001
Thalamo-parietal (T_PAR)	L	2.31	0.041	-4.10	0.0001
	R	2.78	0.005	-4.55	<0.0001
Thalamo-postcentral (T_POSTC)	L	3.21	0.002	-5.58	<0.0001
	R	4.10	<0.0001	-5.42	<0.0001
Thalamo-precentral (T_PREC)	L	3.78	0.0002	-6.40	<0.0001
	R	4.46	<0.0001	-6.56	<0.0001
Thalamo-prefrontal (T_PREF)	L	2.77	0.007	-6.19	<0.0001
	R	3.17	0.001	-6.43	<0.0001
Thalamo-premotor (T_PREM)	L	4.29	<0.0001	-8.22	<0.0001
	R	5.32	<0.0001	-7.52	<0.0001
Uncinate fascicle (UF)	L	3.65	0.0003	-6.99	<0.0001
	R	3.70	0.0002	-6.32	<0.0001

Supplementary Table 3. Cross-sectional study: white matter tracts differences between individuals with Down syndrome and healthy controls.

**Effective longitudinal relaxation time (R1)**		**side**	**Cohen's *d***	**p $_{\text{uncorrected}}$**
**6M > BL**	*PlanumTemporale*	R	1.63	0.030

Supplementary Table 4. Longitudinal study: differential grey matter R1 changes in Down syndrome after 6 months of GnRH therapy compared to changes in healthy controls.

x = MoCA delta				
**Magnetization transfer saturation (MTsat)**		**side**	**Std-β**	**p FWE**
Tracts	*Superior longitudinal fasciculus I (SLF_I)*	L	-0.367	0.044
	Thalamo-postcentral (T_POSTC)	R	-0.309	0.043
**Effective longitudinal relaxation time (R1)**		**side**	**Std-β**	**p FWE**
Tracts	*UF*	L	-0.196	0.004
**x_1 = MoCA baseline, x_2 = MoCA delta**				
**Magnetization transfer saturation (MTsat)**		**side**	**Std-β**	**p FWE**
Tracts	*Rostrum (CC3)*	-	-10.00	0.010
	Anterior midbody (CC4)	-	-15.96	0.001
	Superior longitudinal fasciculus II (SLF_II)	L	-8.63	0.020
	Superior longitudinal fasciculus I (SLF_I)	L	-10.02	0.010
		R	-9.02	0.017
	Superior thalamic radiation (STR)	R	-7.60	0.037
	Thalamo-postcentral (T_POSTC)	L	-8.18	0.026
		R	-7.12	0.050
	Thalamo-precentral (T_PREC)	L	-10.97	0.006
		R	-8.28	0.025
	Thalamo-premotor (T_PREM)	L	-8.24	0.025
		R	-11.70	0.005
**Effective longitudinal relaxation time (R1)**		**side**	**Std-β**	**p FWE**
tracts	*Uncinate fasciculus (UF)*	L	-15.95	0.001

Supplementary Table 5. Post hoc analysis results of associations between longitudinal changes in cognitive performance and brain anatomy confined to the Down syndrome group.
